# Isothermal Recombinase Polymerase Amplification (RPA) of *E. coli* gDNA in Commercially Fabricated PCB-Based Microfluidic Platforms

**DOI:** 10.3390/mi12111387

**Published:** 2021-11-12

**Authors:** Maria Georgoutsou-Spyridonos, Myrto Filippidou, Georgia D. Kaprou, Dimitrios C. Mastellos, Stavros Chatzandroulis, Angeliki Tserepi

**Affiliations:** 1 Institute of Nanoscience and Nanotechnology, NCSR-Demokritos, Patriarchou Gregoriou E’ and 27 Neapoleos Str., Aghia Paraskevi, Attiki, 15341 Athens, Greece; m.georgoutsou-spyridonos@inn.demokritos.gr (M.G.-S.); m.filippidou@inn.demokritos.gr (M.F.); gdkaprou@gmail.com (G.D.K.); s.chatzandroulis@inn.demokritos.gr (S.C.); 2 Institute of Nuclear & Radiological Sciences and Technology, Energy & Safety, NCSR-Demokritos, Patriarchou Gregoriou E’ and 27 Neapoleos Str., Aghia Paraskevi, Attiki, 15341 Athens, Greece; mastellos@rrp.demokritos.gr

**Keywords:** PCB technology, DNA amplification, RPA, microfluidics, microheaters, *E. coli*, molecular diagnostics

## Abstract

Printed circuit board (PCB) technology has been recently proposed as a convenient platform for seamlessly integrating electronics and microfluidics in the same substrate, thus facilitating the introduction of integrated and low-cost microfluidic devices to the market, thanks to the inherent upscaling potential of the PCB industry. Herein, a microfluidic chip, encompassing on PCB both a meandering microchannel and microheaters to accommodate recombinase polymerase amplification (RPA), is designed and commercially fabricated for the first time on PCB. The developed microchip is validated for RPA-based amplification of two *E. coli* target genes compared to a conventional thermocycler. The RPA performance of the PCB microchip was found to be well-comparable to that of a thermocycler yet with a remarkably lower power consumption (0.6 W). This microchip is intended for seamless integration with biosensors in the same PCB substrate for the development of a point-of-care (POC) molecular diagnostics platform.

## 1. Introduction

The accommodation of conventional laboratory processes in microfluidic platforms fabricated using well-established microfabrication technology has drawn great attention and led to the development of the so-called lab-on-a-chip (LOC) devices. Typical advantages of microfluidic systems include the possibility to use very small quantities of expensive reagents and scarce samples, to perform high-resolution, precise, and sensitive detection, and to reduce the analysis time and cost [1]. Such systems are capable of performing a great variety of laboratory processes, such as sample purification and enrichment [2,3,4], reagent mixing [5,6], ultra-fast thermal cycling required in many biochemical reactions [7], as well as the detection of reaction products [8,9], which is of utmost importance. To achieve the above, LOC devices typically integrate microfluidic components, electrical driving circuits, and sensors into the same, usually hybrid, platform [8,10,11,12].

A drawback of such hybrid LOC platforms is that different substrate materials are used, and thus, different technologies need to be implemented for their fabrication, rendering the integration cumbersome and costly, thus hindering their commercial exploitation. Therefore, integrated LOC devices fabricated seamlessly on a single substrate are desirable and a fabrication technology amenable to mass production is sought to enhance the potential for commercialization of microfluidic-based diagnostic devices [8,13].

Nucleic acid analysis is an increasingly important tool for a wide variety of biochemical applications, such as molecular diagnostics, and as such, it is included as a step in many LOC devices and systems [14,15]. The uniqueness of nucleic acid sequences allows for the detection of biological agents with a high degree of specificity. However, since the concentration of nucleic acids in biologically derived analytes is low, their concentration must be amplified to be effectively used as a detection tool. This can be achieved by nucleic acid amplification (NAA) methods, such as the polymerase chain reaction (PCR), which is considered the gold standard in molecular diagnostics, as well as convenient isothermal amplification methods (including the helicase dependent amplification (HDA), the loop-mediated amplification (LAMP), and the recombinase polymerase amplification (RPA)), which are preferred for their simplified protocols and the elimination of thermocycling, which is accommodated in many LOC devices [16,17].

The implementation of (rigid or flexible) printed circuit board (PCB) substrates and the relevant technology for the realization of microdevices for NAA has been described in [18,19,20,21,22], proposing PCB technology as a convenient platform for integrating electronics and microfluidics, thus facilitating the introduction of such devices to the market, thanks to the inherent upscaling potential of the PCB industry and the well-established PCB technology around the world. PCB-based microdevices for static chamber [22] and continuous flow [21] PCR, as well as isothermal HDA-based DNA amplification [20] eliminating thermal cycling, have been described in the past. Amongst various isothermal NAA methods, RPA has attracted much attention due to its minimal sample preparation, increased sensitivity, specificity, robustness, and low cost, thus rendering it a perfect candidate for diagnostics in resource-limited settings [23]. Despite its advantages, an integrated PCB-based RPA microchip has not been yet reported.

In more detail, RPA is a NAA method carried out under isothermal conditions, thus not requiring thermocycling. Its main advantage is its low operational temperature near body temperature (37–42 °C), combined with its simplicity (minimal sample preparation, it can be carried out directly in serum, urine, as well as in the presence of known PCR inhibitors), sensitivity (down to 1–10 target DNA copies), and speed (5–20 min) [24,25]. The RPA method, first introduced by Piepenburg et al. [26], couples the isothermal recombinase-driven primer binding to the template DNA with the strand-displacement DNA synthesis. RPA is a compelling alternative to PCR, addressing the rapid detection of various pathogenic agents such as bacteria [23,27,28,29,30,31], viruses [32,33,34], parasites [35], and fungi [36]. In most of the previous works, the emphasis was on the development of POC diagnostic platforms for performing microbial analysis at the point-of-need; however, little attention was paid to the mass production of integrated chips, to allow for the massive deployment and adoption of microfluidics in the diagnostics market. Nevertheless, situations imposed by a pandemic such as COVID-19 provide additional motivation for the rapid development of new diagnostic microdevices [37] that are fast and massively fabricated by an established industry.

In this work, an RPA-on-PCB microdevice for performing DNA amplification was designed, commercially fabricated, and validated for performing DNA amplification of fragments of two genes of *E. coli*, which is a common Gram-negative bacterium that normally resides within the intestinal microbiota of humans. However, certain highly virulent *E. coli* strains can cause serious health conditions such as urinary tract infections (UTIs), respiratory illness, and pneumonia. While UTIs can result from both Gram-negative and Gram-positive bacterial expansion, the majority of UTI cases are attributed to *E. coli* strains [38]. Therefore, *E. coli* serves as a model pathogen for addressing UTI pathogenesis and developing relevant diagnostics. In this work, target gene-specific primers were designed and validated, while the RPA performance of the microdevice was compared to that of a conventional thermocycler. This microdevice is intended to be integrated in the same PCB substrate with biosensors for the development of a microdevice serving as a POC molecular diagnostics platform.

## 2. Materials and Methods

### 2.1. Design and Fabrication of an RPA-on-PCB Microchip

For the on-chip evaluation of the RPA amplification, a microchip was designed and fabricated on PCB, as the PCB technology allows for low-cost and standardized mass production, while the PCB substrate enables all the electrical connections required for the operation of the device. The chip was designed using an open source software, Kicad, whereas it was fabricated by a PCB manufacturing company, Eurocircuits (https://www.eurocircuits.com).

**Figure 1:**
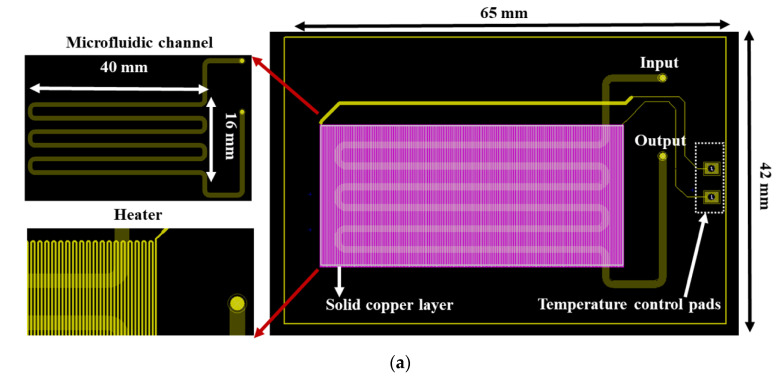

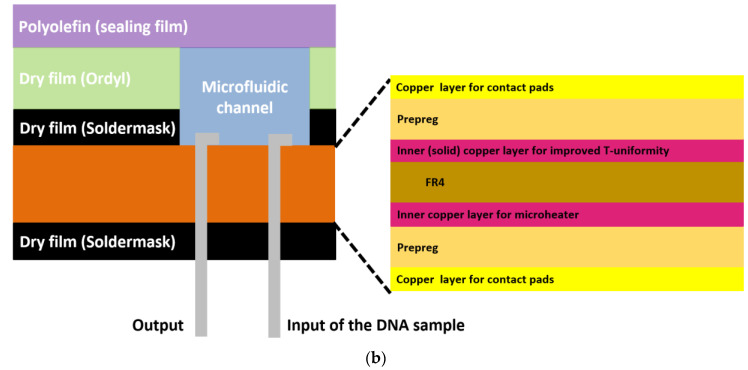
(**a**) RPA-on-PCB chip design for DNA amplification. The meandering microfluidic channel, the microheater with its electrical pads, as well as a solid copper layer beneath the microchannel for optimum temperature uniformity are depicted. (**b**) Schematic cross-sectional view of the RPA-on-PCB chip.

In particular, the chip dimensions were as follows: thickness: 1.55 mm, length: 65 mm, and width: 42 mm, and it consisted of a meandering microfluidic channel (occupying an area of 16 × 40 mm^2^, Figure 1a), to minimize the chip footprint, on one side of the PCB substrate and a copper (Cu) microheater on the other side. The microchannel was patterned on a laminated photosensitive dry film (Figure 1a), and it had dimensions of 300 mm, 1 mm, and 100 μm for length, width and height, respectively, and a volume of 30 μL. Smaller volumes were also possible with the implementation of thinner photosensitive dry films. The microchannel was partly commercially fabricated on the solder mask layer, which was supplied together with the PCB from the PCB manufacturer (Eurocircuits, Kettenhausen, Germany), while for increased microfluidic channel height, the solder mask layer was combined in house with an ORDYL SY 300 film purchased from Resistechno. A cross-sectional view of the device is shown in Figure 1b. The microheater was implemented in the inner 18 μm-thick Cu layer of the PCB substrate (meandrous yellow tracks in Figure 1a) in the area below the microfluidic channel to allow for proper heating of the DNA amplification cocktail during the amplification, and it was isolated from the microfluidic channel by insulating layers. Furthermore, in order to optimize the temperature uniformity in the chip area where the microchannel was built [22], a solid Cu layer (magenta rectangle in Figure 1a) was formed on the inner 18 μm-thick Cu layer exactly under the microchannel. In order to provide for sample input and output, two through holes were opened at the beginning and end of the microchannel. Finally, the microchannel was sealed in house with a 100 μm polyolefin film (StarSeal from STARLAB) coated on one side with a silicone adhesive (PCR-compatible) in order to provide a strong adhesion even at elevated temperatures.

### 2.2. Temperature Control

Control of the microheater temperature was achieved using a temperature control unit providing the voltage needed across the resistive microheater, whilst a 100 mOhm resistor, placed in series with the microheater, was used to measure the current flowing through. Thus, the controller measures in real time the resistance of the microheater to derive its operating temperature through the temperature coefficient of resistance for copper (i.e., using the microheaters as temperature sensors) [21], and the resulting value was used in a proportional–integral (PI) feedback control loop to stabilize the temperature of the microheater at the desired set point.

### 2.3. Biological Assays

In this work, RPA was optimized and performed on a PCB-based microfluidic chip for the amplification of *E. coli* DNA. Toward this end, *E. coli* bacterial cultures were performed, followed by DNA extraction. Appropriate primers were also designed, and amplification methods were performed both in a conventional thermocycler and on chip. The assays are described in detail below.

#### 2.3.1. Bacterial Culture 

All experiments were performed with the strain of *Escherichia coli* TOP10. *E. coli* was grown overnight in Luria–Bertani (LB) medium (37 °C, 200 rpm); bacterial cells were inoculated in a fresh cultural medium (1:1000). Bacterial cultures were grown until mid-late exponential phase in liquid media. Cells were centrifuged and resuspended in 0.9% NaCl. Optical density was measured corresponding to different cell concentrations, 10^3^–10^7^ CFU/mL. To quantify the concentration of bacteria in saline solution (internal control), viable spread plate counts were determined by serial dilution plating on solid LB agar media.

#### 2.3.2. DNA Extraction: Chemical and Thermal Lysis

Chemical Lysis: The genomic DNA (gDNA) from the bacterial cultures was extracted using enzymatic lysis buffer containing Proteinase K, RNase, Lysozyme, and SDS. The DNA was purified using phenol/chloroform solution and precipitated by ethanol. The concentration and the purity of the DNA samples were measured using a nanodrop spectrophotometer (Nanodrop 1000, ThermoFisher Scientific, Paisley, UK). Each DNA sample was standardized to 50 ng/μL and stored at −20 °C until use.

Thermal Lysis: First, 1 ml of *E. coli* culture in 0.9% NaCl was incubated for 10 min at 95 °C in thermoblock (Digital Dry Bath, Biorad). The sample was centrifuged (3000 rpm, RT °C 10 min), and the supernatant containing gDNA of *E. coli* was used as the cell lysate in the reactions.

#### 2.3.3. Primer Design

According to the instructions for the RPA kit (TwistDX, Cambridge, UK, www.twistdx.co.uk/docs/default-source/RPA-assay-design) [39], RPA requires a different parameter design from that needed for PCR analysis (for example, a longer length of the primer, approximately 30–35 nucleotides). In addition, there are no melting temperature requirements for the design of RPA primers because hybridization and elongation are achieved by enzymes and are not induced by temperature [24]. Therefore, the primer design should consider multiple factors, including hairpin structure, mismatch, primer dimer, and amplification efficiency. In the present study, two target-specific primer pairs were designed for the amplification of a 210 bp fragment of the yBBW gene and a 176 bp DNA fragment of the malB gene. These primers were designed based on previously published primer sequences for the same *E. coli* genes and modifications including the extension of primer length. At the same time, the designed primers are also suitable for PCR amplification. The primer sequences used in this study are given in Table 1. All oligonucleotides were synthesized by Metabion International AG (Planegg, Germany) and purified by high-pressure liquid chromatography (HPLC). Oligonucleotides were delivered as dry, lyophilized powder, were dissolved in nuclease-free water at a concentration of 100 μΜ, and stored at 20 °C, in the dark.

#### 2.3.4. Amplification Methods: PCR and RPA

For PCR, the KAPA2G Fast ReadyMix (KapaBiosystems, Wilmington, MA, USA) kit was used according to the supplier’s instructions (https://www.kapabiosystems.com/product-applications/products/pcr-2/kapa2g-fast-pcr-kits/). KAPA 2G Fast DNA polymerase was mixed with 5 pmoles of each primer. Each 25 µL PCR reaction was supplemented with 1 µL of DNA template with a concentration of 1 ng/μL. DNA fragments encoding ybbW and malB genes were amplified, and each reaction contained 12.5 μL Buffer. Purified genomic *E. coli* TOP10 DNA (1 ng) was used as the template. The PCR protocol used in the thermocycler consisted of 3 steps of 10 s denaturation at 95 °C—10 s annealing at 60 °C (ybbW) and 55 °C (malB)—10 s extension at 72 °C, repeated 30 times. Subsequently, the PCR products were loaded on agarose gel (2%) stained with ethidium bromide and visualized with an ultraviolet (UV) visualizer.

RPA reactions were accomplished using commercially available RPA reagent kits provided in the TwistAmp Exo Kit, available from TwistDX Ltd. (Cambridge, UK). Each primer was used at a final concentration of 10 µM; the final volume was 25 μL. Purified genomic *Escherichia coli* TOP10 DNA (1 ng) was used as the template. RPA reactions were performed at 39 °C, for 30 min, in 50 µL volumes consisting of 29.5 μL of rehydration buffer, 2.4 µL of each primer (10 µM), a tube of lyophilized enzymes diluted in 16 μL dH_2_O, and 1–2 ng of DNA template or 1 μL cell lysate. After thorough mixing, 2.5 µL of 280 mM MgOAc was added into the reaction system. Before the diluted enzymes were added to the reaction mixtures, they were irradiated with UV light in order to degrade and inactivate any DNA contamination, which could cause a false-positive amplification, as described previously [40]. In miniaturized amplification assays, RPA reactions were carried out for 10–30 min, in 12.5, 25, and 50 μL volumes. The experiments were executed in a conventional thermocycler (T-Personal 005-552, Biometra) and in a static PCB-based microdevice. RPA products were purified from enzymes and proteins using a NucleoSpin^®^ Gel and PCR Clean-up kit (MACHEREY-NAGEL GmbH & Co, Düren, Germany). The purification procedure was necessary to visualize DNA bands in agarose gel electrophoresis. Alternatively, RPA products can be heat treated (5 min at 95 °C).

## 3. Results and Discussion

For the validation of the RPA-on-PCB microdevice, an isothermal protocol was optimized and followed for fast and efficient DNA amplification. Amplification reactions were carried out in the RPA microdevice and on a conventional thermocycler for comparison purposes. The results are presented and discussed in the following sections.

### 3.1. Selection of Primers for E. coli DNA Amplification

*Escherichia coli* are a common, large, and diverse group of bacteria found in the environment, food, and intestines of people and animals. Although most strains of *E. coli* are harmless, some can cause diarrhea, while others are responsible for 80 to 90% of the urinary tract infections. The RPA was selected as the amplification method because it is an isothermal one, thus avoiding thermocycling, while in addition, it can reduce analysis time much below 60 min. The *E. coli* strains have substantially diverse and multiple sequences. Alignment analysis of *E. coli* genomes reveals that the selection of universal and specific genetic targets in this bacterium is a great challenge. The target genes should be evolutionarily conserved to be found in all strains. In addition, it is essential to avoid the co-amplification of material extracted from other bacteria species or close related organisms. Therefore, the primer sequences should be carefully selected and designed to have 100% exclusivity and inclusivity.

A novel RPA assay was designed for the detection of two target genes ybbW and malB that, after in silico analysis, comply with the above requirements [41,42,43]. According to Walker et al. [41], the ybbW gene is part of the core genome (existing in >95% of all sequenced strains) of the *E. coli* offering great inclusivity (100%) and exclusivity (100%) within *E. coli*, whereas the malB gene is conserved across different *E. coli* lineages [44]. The ybbW gene sequence codes for a putative allantoin permease involved in the transport and metabolic conversion of allantoin, which is a metabolic intermediate that can serve as a source of nitrogen for bacterial cells under nutrient-limiting conditions (https://www.uniprot.org/uniprot/P75712). The malB gene sequence is derived from a large genomic region of *E coli* coding for a set of gene products (malB operon) involved in the transport, utilization, and metabolic turnover of maltose within bacterial cells. It is highly conserved among bacterial species. The region of the malB operon selected for *E coli*-specific primer design corresponds to a region that is discrete from other bacterial species and highly conserved among most *E coli* strains [45].

**Figure 2:**
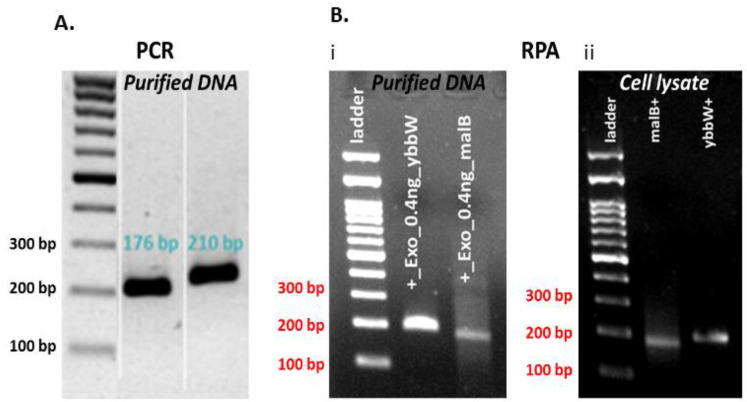
(**A**) Agarose gel electrophoresis (2%) of PCR products amplified by specific primer pairs for ybbW and malb genes with purified gDNA *E. coli* TOP10 (1 ng—2 × 10^5^ copies DNA) template. (**B**) Agarose gel electrophoresis (2%) of RPA products amplified by target genes primer pairs with purified gDNA *E. coli* TOP10 (1 ng) and cell lysate (1 μL—2 × 10^7^ copies DNA) (thermal lysis) template.

One set of novel primers was used for the successful amplification of each target gene. In addition, alignment studies of *E. coli* genes were completed using sequence information from the National Center for Biotechnology Information (NCBI) Genbank database. The primers were designed to be suitable for the RPA method and specific for amplification of the selected target genes (first, with a length 30–35 bp—applicable only for the ybbw, not for the malB primer set—and <45 bp, second, with a content in GC nucleotides >30% or <70%, and third, with amplification products 80–400 bp). Primer sequences were selected with the aid of Primer 3, and their selectivity for *E. coli* was determined using the Primer-BLAST algorithm. The primer analysis for dimers and hairpins was performed using the software offered by Integrated DNA Technologies (IDT, https://eu.idtdna.com/pages). The primers were tested in addition to RPA by the polymerase chain reaction (PCR) method in the thermal cycler. PCR was performed in a 25 μL reaction volume for DNA samples that comprised 1 μL purified DNA template (1 ng) and 10 µM primers. RPA was performed in a 50 μL reaction volume for purified 1 µL DNA or 1 μL cell lysate and 10 µM primers. The DNA amplification was verified via agarose gel electrophoresis. Figure 2 indicates that both primer pairs have excellent specificity for the target genes in both amplification methods, PCR and RPA. In more detail, both ybbW and malB genes are amplified with high efficiency and specificity in PCR, while ybbW is amplified more efficiently than malB in RPA (Image J analysis of the image in Figure 2B (i) indicates a four times higher fluorescence signal for ybbW). For this reason, from this point on in this work, the ybbW gene will be used in RPA for amplification of *E. coli* gDNA. In addition, images in Figure 2B (i) and (ii) indicate that RPA amplifies equivalently purified gDNA and gDNA lysed from *E. coli* cells; therefore, RPA robustness is demonstrated also in this work for requiring minimal sample preparation.

### 3.2. RPA Protocol and Optimization for On-Chip DNA Amplification

Point-of-care genetic diagnostics depend on the miniaturization of both the sample processing and the NAA devices. First, the heating at microscale level is supplied closer to the sample and is applied to a smaller thermal mass than in macroscale systems; this decreases the power consumption as well as the total reaction time [46], providing a faster time-to-result [16]. Furthermore, the miniaturization reduces the volume of the required amplification reagents, which is vital in amplification reactions in which costs are prohibitive to widespread use. Finally, miniaturization enables higher sensitivity and minimizes the risk of sample contamination. In isothermal enzymatic methods such as RPA, in vitro DNA synthesis occurs at a constant reaction temperature (39 °C), and hence, there is no need for an expensive thermal cycling instrument. However, several other factors are essential in performing RPA on a chip (with a static chamber), including the reaction volume and the amplification time.

**Figure 3:**
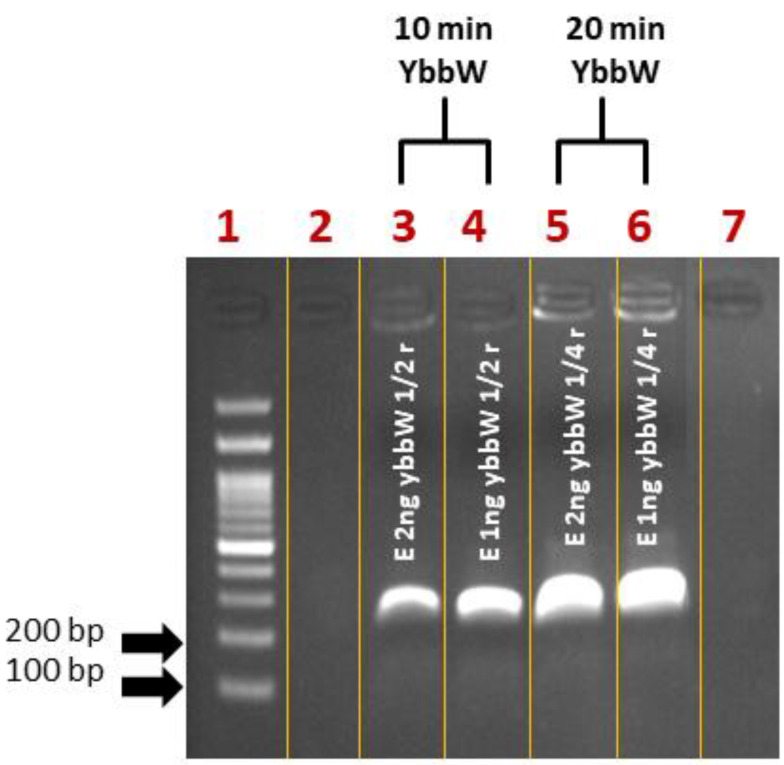
Agarose gel (2%) electrophoresis image of RPA reactions using ybbW primers and gDNA *E. coli* TOP10 (1–2 ng) as a template. The initial reaction was divided into different fractions before the amplification. The original TwistDx assay was performed for shorter time (Lane 3–4: 10 min and Lane 5–6: 20 min) and smaller sample volumes (Lane 3–4: 25 μL and Lane 5–6: 12.5 μL) than those recommended by the manufacturer. The performance of RPA was considered satisfactory.

First, control experiments for RPA miniaturization were performed on a thermal cycler. The DNA amplification was verified via agarose gel electrophoresis. Initially, samples were prepared as recommended by the kit manufacturer in a final volume of 50 μL/reaction. Then, they were divided into smaller fractions (1/2: 25 μL, ¼: 12.5 μL), as convenient sample volumes that can be safely loaded on PCB chips are 25 μL and 12.5 μL, compatible with fabricated microchannel volumes. Amplification reactions were performed for 30 min at 39 °C. For reducing the time-to-result, RPA reactions were also performed for 10 and 20 min. Figure 3 summarizes all RPA results from the cycler and indicates that RPA works with a satisfactory efficiency both in lower volumes (12.5 μL) and shorter time (10 min) than the kit manufacturer recommends, however with a lower amplification efficiency at shorter time (10 min).

### 3.3. Characterization of the RPA-on-PCB Microdevice

**Figure 4:**
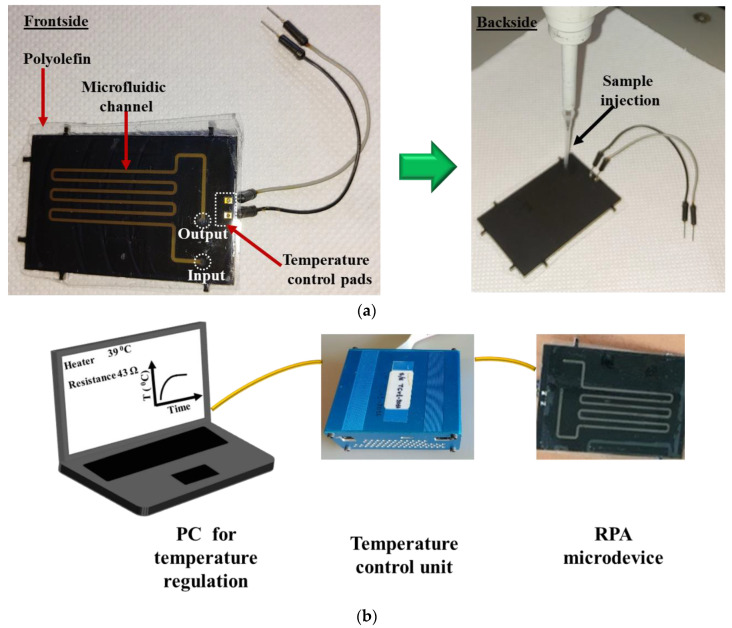
(**a**) Image of the frontside and backside of the RPA-on-PCB chip ready for use. The microfluidic channel and the sealing film (polyolefin) are depicted (left). Image of the backside of the device during the introduction of a RPA solution in the microchannel (right). (**b**) Schematic representation of the experimental set-up, comprising the RPA-on-PCB chip, the temperature control unit, and the PC with the user interface.

Figure 4a depicts the front (left) and the back (right) side of a fabricated RPA-on-PCB microdevice, while Figure 4b depicts the experimental set-up used for the evaluation of the RPA microdevice, comprising, in addition to the chip, the custom-made temperature controller unit and a laptop to facilitate user interfacing. The set-up was simplified by using a pipette (Figure 4a, right) for introducing (and collecting) the sample to the chip.

**Figure 5:**
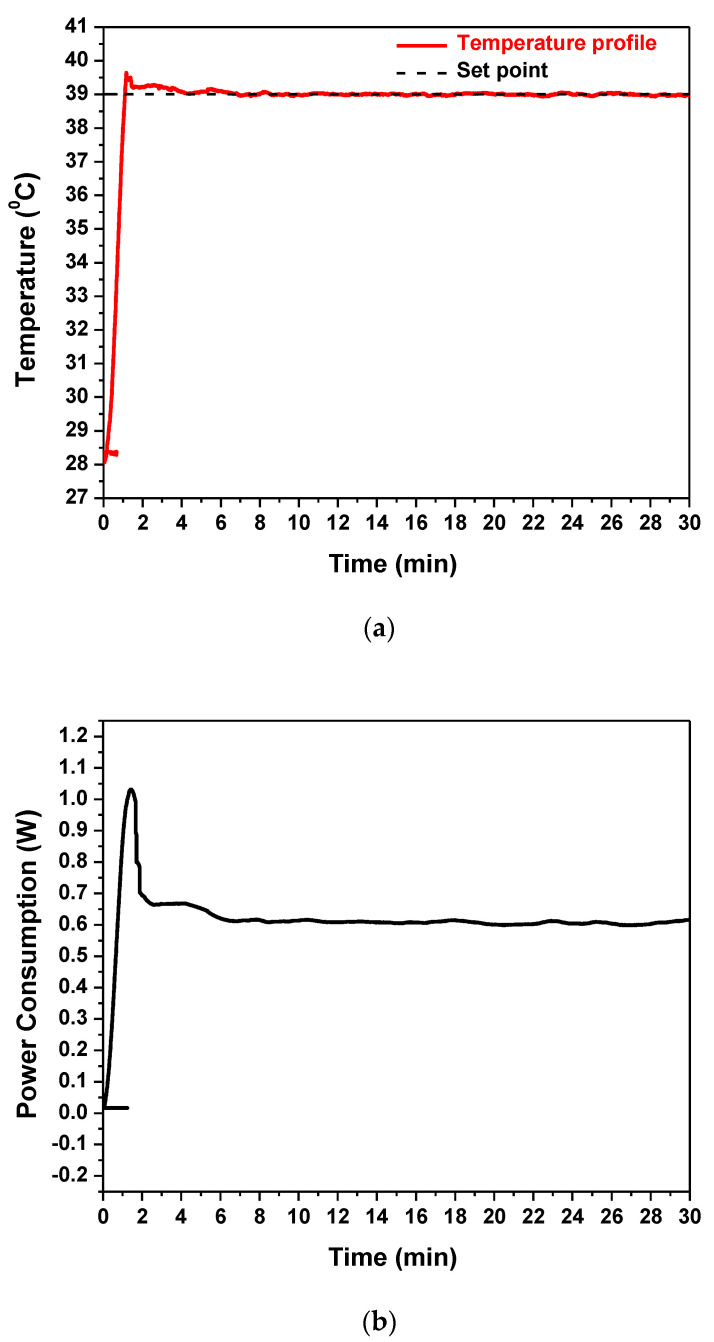
(**a**) The temperature profile of the embedded Cu microheater after heating up to set-point temperature and (**b**) its power consumption.

The embedded Cu microheater of the RPA-on-PCB chip was measured to have a resistance R_0_ equal to 43 Ohm (measured at 25 °C), while the voltage, the current, and the power consumption were recorded during operation, to achieve and stabilize the temperature at the set point by means of the temperature controller. Figure 5a illustrates the temperature profile (red line) recorded by the temperature controller. The diagram indicates that after approximately 1.5 min, the temperature of the microheater reached the desirable set point (39 °C) starting from 28 °C and achieved stabilization at the set point within 5 min, with minimal fluctuations during the entire operation (30 min). In Figure 5b, the power consumption of the chip during operation is shown. After initial heating up from 28 °C, the microheater reached the set-point temperature (the current supplied was approximately 0.12 A), where the average power consumption was stabilized at 0.6 W. This power consumption is, as expected, smaller than that reported in continuous flow microPCR devices realized on PCB (2.7 W [21]) and far smaller than the power consumption of conventional thermocyclers (typically 500 W).

### 3.4. Validation of the RPA-on-PCB Microdevice

**Figure 6:**
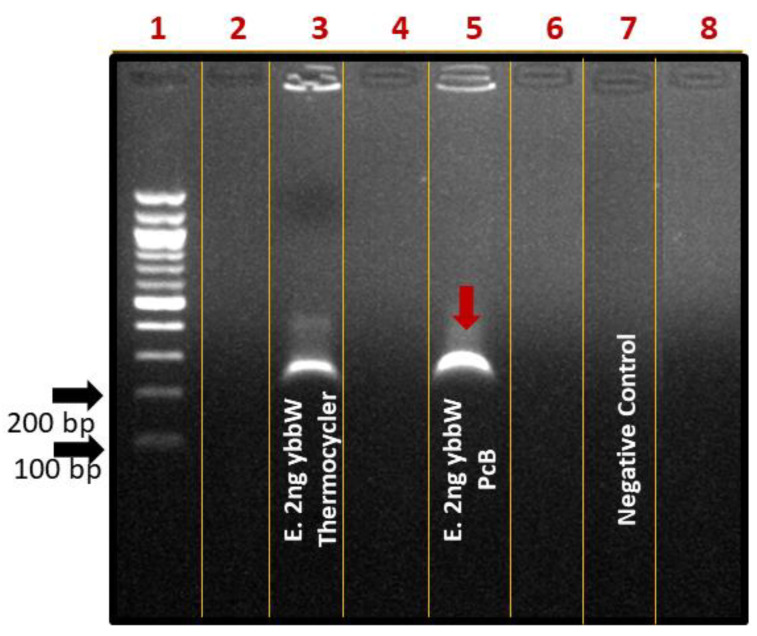
Agarose gel (2%) electrophoresis image of RPA reactions using ybbW primers and gDNA of *E. coli* TOP10 (2 ng) as a template. Lane 1: DNA ladder, Lane 3: positive control-ybbW amplified RPA product, RPA reaction on thermocycler, Lane 4: ybbW amplified RPA product, reaction on PCB chip, Lane 7: negative control-no gDNA, reaction on thermocycler.

For performing on-chip RPA, the fabricated RPA-on-PCB microchip was connected to a custom-made temperature controller (Figure 4b) that senses the microheater’s temperature, while on the other hand, it regulates the voltage across the microheater resistance to allow for precise control of the amplification temperature (39 °C). Before using each PCB microdevice for the first time, a washing step using ethanol was applied. For subsequent uses of the same device, an oxygen plasma step was applied to remove any biomolecule adsorbed on the microchannel surface. At this point, a 25 μL RPA solution containing *E. coli* DNA was introduced in the chamber using a micropipette (Figure 4a), and the temperature was maintained at 39 °C for 30 min, for performing DNA amplification. Simultaneously, a duplicate sample was amplified in the thermocycler as the positive control of the reaction. The amount of purified gDNA of *E. coli* TOP10 that was used as the reaction template was 2 ng. Negative control was also performed, where all reagents were added in the cocktail except for bacterial DNA. At the end of the experiments, the samples were collected by a micropipette and were cleaned up via thermal treatment. Amplification of the ybbW gene target was verified via gel electrophoresis (Figure 6), indicating a high amplification efficiency of the reaction performed on the PCB chip. In fact, analysis via Image J indicates slightly higher amplification on chip compared to that on the cycler. Thus, the results clearly demonstrate that the amplification of the ybbW gene target at 210 bp was successfully achieved in the developed RPA-on-PCB microdevice, with amplification efficiency well-comparable to that of a conventional thermocycler.

The capability of PCB-based chips, similar to the present one, to perform PCR either in continuous flow or in static chamber microdevices has been demonstrated in the past [21,22]. The objective of this work was the demonstration of an RPA isothermal amplification as a simplified method not requiring thermocycling that is mostly appropriate for POC use.

## 4. Conclusions

In this article, we describe the development of a simple, low-cost microfluidic chip commercially fabricated for the first time on PCB, incorporating on the same PCB substrate a microchannel and resistive microheaters that are capable of performing RPA efficiently. The microchip was validated for achieving DNA amplification of two target genes of *E. coli*, which is a common bacterium potentially responsible for urinary tract infections, respiratory illness, and pneumonia. Specific primers were validated, while the RPA performance of the microchip was found to be well-comparable to that of a conventional thermocycler, yet with a remarkably lower power consumption. This microchip is intended in the near future to be seamlessly integrated with biosensors in the same PCB substrate for the development of a point-of-care molecular diagnostics platform.

## Figures and Tables

**Table 1:** Primers designed in this study.

Name	Oligo Sequence	Length	GC%	Tm (°C)
FybbW	5′- TGA TTG GCA AAA TCT GGC CGG GAT TTT TAA CT-3′	31	38.7	61
RybbW	5′-GAA ATC GCC CAA ATC GCC ATA CCG CCG AAA AC-3′	32	53.1	66
FmalB	5′-GGA TAT TTC TGG TCC TGG TGC CGG-3′	24	58.3	62
RmalB	5′-TTT TCG ATG TGC GTT TAG CGC AGA-3′	24	45.8	60
